# Accelerometric Changes before and after Capacitive Resistive Electric Transfer Therapy in Horses with Thoracolumbar Pain Compared to a SHAM Procedure

**DOI:** 10.3390/ani10122305

**Published:** 2020-12-05

**Authors:** David Argüelles, Mireya Becero, Ana Muñoz, Aritz Saitua, Toni Ramón, Eduard Gascón, Antonia Sánchez de Medina, Marta Prades

**Affiliations:** 1 Department of Animal Medicine and Surgery, School of Veterinary Medicine, University of Córdoba, 14004 Córdoba, Spain; darguellescap@gmx.es (D.A.); tsmedina189@hotmail.com (A.S.d.M.); 2 Veterinary Teaching Hospital, School of Veterinary Medicine, University of Córdoba, 14004 Córdoba, Spain; mireyabece_1394@hotmail.com (M.B.); aritz_sp91@hotmail.com (A.S.); 3 Equine Sport Medicine Center CEMEDE, School of Veterinary Medicine, University of Córdoba, 14004 Córdoba, Spain; 4 Physiotherapy and Osteopathy Equine Practice, 08913 Barcelona, Spain; toni@fisiovet.com; 5 Department of Animal Medicine and Surgery, School of Veterinary Medicine, Autonomous University of Barcelona, 08193 Bellaterra, Spain; eduard.gascon@uab.cat (E.G.); marta.prades@uab.cat (M.P.)

**Keywords:** accelerometry, capacitive resistive electrical transfer, electrophysical agents, horse, thoracolumbar pain

## Abstract

**Simple Summary:**

Capacitive resistive electric transfer (CRET), a radiofrequency at 448 kHz, has been shown to result in increased muscle oxygenation and flexibility in the quadriceps muscle of human beings. In this study, 18 sport horses with thoracolumbar pain were divided into two homogenous groups: CRET (*n* = 9), subjected to four sessions of CRET during two consecutive weeks and SHAM (*n* = 9), subjected to the same sessions but with the device off. Clinical examination and accelerometry were made at the beginning and at the end of the study. A Mann-Whitney test and a Wilcoxon matched pair test were used to compare between SHAM and CRET groups and before and after the intervention, respectively. CRET horses showed a reduction of 1 degree in thoracolumbar pain (*p* = 0.002) and of 2 degrees in epaxial muscle pain (*p* = 0.03). SHAM horses had a reduction of 1 degree in thoracolumbar pain (*p* = 0.01). CRET horses presented increased dorsoventral power at walk and trot (*p* < 0.002), probably reflecting increased dorsoventral movement and flexibility. Such changes were not found in SHAM horses. No changes were found in the dorsoventral displacement of the center of gravity in either group. Our study demonstrated that sport horses with mild to moderate thoracolumbar pain could improve clinically and biomechanically after CRET therapy.

**Abstract:**

Capacitive resistive electric transfer (CRET), a radiofrequency at 448 kHz, increases flexibility in quadricep muscles of human athletes. To assess whether CRET would result in clinical and biomechanical improvements in horses with thoracolumbar pain, 18 sport horses were divided into two groups: CRET (*n* = 9), subjected to four CRET sessions, during two consecutive weeks, and SHAM (*n* = 9), subjected to the same procedure with the device off. Clinical examination and accelerometry were performed before and after the four sessions. During the study, horses were in training and in active competition, and did not receive any other treatment. Mann-Whitney and a Wilcoxon matched pair tests were used to compare between the SHAM and CRET groups and before and after the intervention, respectively. CRET horses showed increased dorsoventral (*p* < 0.002), mediolateral and total power (*p* < 0.01) after the intervention, suggesting increased back flexibility. SHAM horses did not show any of these modifications after the intervention. No changes were found in the dorsoventral displacement of the gravity center in either group. Thoracolumbar pain decreased one degree after CRET (*p* = 0.002), and it did not change after SHAM. Epaxial muscle pain decreased two degrees after CRET (*p* = 0.03) and one degree after SHAM (*p* = 0.01). These results reflected that CRET therapy would increase back flexibility and decrease thoracolumbar and epaxial pain.

## 1. Introduction

Thoracolumbar pain and associated neuromuscular dysfunction are common clinical entities in the athletic horse [1,2,3]. A great variety of specific disorders may cause thoracolumbar pain in horses, and affect several anatomical structures in the area, i.e., bones, muscles, joints and ligaments, although the most common disorders are the impingement of the dorsal spinous processes and osteoarthritis of the articular facets [4,5,6]. Regardless of the source/s of back pain, some authors have described the pathophysiological pattern of back pain in horses by extrapolating the knowledge and consequences of low back pain in human beings. According to Stubbs et al. [4], a bone/joint disease triggers an ipsilateral selective atrophy of the multifidus muscles at the same spinal level, which results in spinal instability and subsequently the epaxial musculature tightens in order to compensate, therefore creating stiffness or hypertonicity [7,8]. The architecture of this muscle as well as its anatomical attachments does not allow the stabilization of the individual joints [9]. Therefore, the elimination of the secondary muscle hypertonicity and spasms may be an important strategy to mitigate chronic pain.

Capacitive resistive electric transfer (CRET) is a non-invasive electrothermal therapy based on the application of radiofrequency electric currents at a range of 400–450 kHz, which pass between an active and an inactive electrode. Due to the electrical resistivity of the tissues, CRET currents could increase temperature in the target organs. Circulating blood dissipates the heat towards the adjacent areas, allowing the temperature of the treated structures to be kept within desired limits, avoiding unwanted tissue hyperthermia [10]. Furthermore, there is scientific evidence suggesting that, at least at the cellular level and in vitro studies, the effect of CRET is not limited to its thermal action. Hernández-Bule et al. [11] (2014) demonstrated in vitro proliferative effects of the CRET currents on adipose-derived stem cells, indicating that lesion repair could be mediated by the stimulation of the proliferation and subsequent differentiation of the stem cells present in injured tissues.

To the authors’ best knowledge, only Tashiro et al. [10] and Yokota et al. [12] have documented the changes in temperature after CRET application in living human beings. Recently, López de Celis et al. [13] measured tissue temperature in the Achilles tendon and musculotendinous junction, but they performed their study in fresh frozen human cadavers. Two similar studies performed on 13 human beings measured the temperature at three different depths: skin or superficial temperature, and deep temperatures at 10 and 20 mm below the superficial skin, in the hamstring muscles [10,12]. A significant increase in temperature was found at each depth, when comparing the application of hot packs. In addition, this rise in temperature resulted in increased concentrations of oxygenated and total hemoglobin concentrations, which were maintained for at least 30 min after therapy. Later, Yokota et al. [14] used the Ely test in twenty-two healthy males to assess the quadriceps muscle flexibility after intense exercise. They observed a significant decrease in the angle of the Ely test (which suggests a decrease in quadriceps flexibility immediately after knee extension exercise), as well as a significant increase in the angle of the anterior pelvic tilt after exercise. These changes were attributable to muscle fatigue and hardness. In the control group (without intervention), significant differences in the Ely test results and pelvic tilt were observed until 30 min after exercise, whereas in the CRET group, these differences were no longer observed, suggesting that quadriceps muscle flexibility returned to the baseline sooner in the CRET group compared to the control group.

The therapeutic effect of CRET therapy in horses has not been objectively evaluated yet. There is only one previous study performed by our research team, which demonstrated that the application of CRET therapy resulted in significant changes in locomotion measured by accelerometry, interpreted as favorable for the sport horse. It was speculated that these effects could have derived from increased muscle temperature, even though this hypothesis could not be proven [15]. In the present study, we evaluated the accelerometric changes in horses with back pain treated with CRET therapy as the only treatment in comparison to a SHAM procedure. With this study, we would like to assess the clinical utility of CRET therapy in the management of equine chronic thoracolumbar pain. We hypothesized that the application of this therapy would result in a greater back flexibility. More specifically, we hypothesized that a CRET intervention compared to a SHAM procedure will lead to increased dorsoventral displacement (DVD) and to a greater power directed to this axis (dorsoventral power, DVP). This hypothesis arises from previous kinematic studies performed in horses with natural back pain, which reported that, in order to alleviate pain, horses experienced back stiffness with a reduction in dorsoventral flexion–extension range of motion and in lateral bending [3,16]. As a second hypothesis, we proposed that horses in the CRET group will show an increase in total power (TP) during the period of study. As a third hypothesis, we think that horses after CRET therapy would present a more regular and symmetric stride, both at walk and at trot.

In addition to the data provided by Wennerstrand et al. [3], the three hypotheses were proposed according to previous results obtained with the same device to what we used in our research in horses with thoracolumbar pain. Aujol et al. [17] described that horses with back pain had increased DVD, DVP, longitudinal power (LP) and stride regularity (REG) after an osteopathic treatment. Some years later, Calvo-Santesmases et al. [18] reported that horses with back pain compared to sound horses had lower DVD, DVP, MLP and TP.

## 2. Material and Methods

This study complies with the current legislation on Bioethics and Biosafety in Europe. The authors proceeded to consult the Bioethics and Biosafety Committee for Experimental Animal of the Autonomous University of Barcelona. We were informed by this Committee that acts of veterinary treatment were excluded from their consent. Furthermore, the rules for proper clinical activities by the veterinarian, established by the Official Council of Veterinary Colleges of Spain, were strictly followed. Moreover, a signed consent from owners or from a responsible of the horses was mandatory in order to be included in the study.

### 2.1. Horses

Horses were selected from a private horse center specialized in training dressage and jumping horses. A total of 80 horses were assessed for participation in the study, and from them, 18 were recruited after applying the established inclusion criteria. These criteria were: (1) to have a complaint of mild to moderate thoracolumbar pain according to the trainer with a duration of at least 3–4 months; (2) to maintain the same training program during the study and to continue in active competitions; (3) to be under the supervision of the same trainers during all of the study; (4) to hold to the study protocol, with the corresponding interventions and the different clinical examinations and accelerometric recordings; (5) not be subjected to any other treatment (systemic or local administration of non-steroid anti-inflammatory drugs,) or other types of treatment, including mesotherapy, acupuncture and chiropractic for at least 4 months before the study; (6) have no evident signs of lameness at the walk and trot, over soft and hard surface, on a straight line and on the lunge. The grade of lameness must not be greater than 1 over 5 in accordance with the American Association of Equine Practitioners (AAEP) scale; (7) absence of radiographic findings in the vertebral joints and ultrasonographic findings in the supraspinous ligament; (8) not having 3 or more radiographic findings attributable to overriding dorsal spinous processes with clinical compatible signs in the same areas.

The 18 horses selected had a mean age of 9.5 ± 2.06 years (range 5–12), they belonged to different breeds (2 Dutch Warmblood, 6 Selle Français, 4 Belgian Warmblood, 2 Holsteiner, 2 Hannoverian, 1 Spanish Sport Horse, 1 Spanish horse) and were of both genders (3 females and 15 males). Four of them were in training for dressage and 14 for jumping. Dressage horses were competing at the Prix St George level and show jumpers competed from 1.20 to 1.40 m. Show jumping horses were trained 5 days per week, jumping 2 days/week and performing other types of exercises 3 days/week. Daily sessions had durations of 40–45 min. Dressage horses were also trained for 5 days/week, with daily dressage exercise sessions of 60–70 min.

The horses were randomly distributed into two groups, after considering two criteria in order to have homogeneous groups. The same number of horses from each type of competition was included in both therapy groups (*n* = 9 horses/group; 2 dressage horses and 7 jumpers) and with a similar scoring of radiographic findings (7 horses with score 1; 2 horses with score 2 for each of the groups). The two groups of horses were subjected to different interventions: group CRET, subjected to CRET therapy and group SHAM, subjected to the same treatment, but with the device without power.

### 2.2. Clinical Examination

Back examination was performed by a board-certified surgeon, a certified equine physiotherapist, following a double-blinded protocol, before the study, when applying the diagnostic criteria for selection within the whole horse population of the center, and after the treatment (CRET and SHAM groups). Horses were evaluated in a quiet stall, without sedation.

Back examination was made according to the protocol described by Trager et al. [6]. In this score, three main characteristics were scored: physical evidence of thoracolumbar and epaxial muscle pain, evaluated by palpation on the midline, directly over the spinous processes and on both sides, over the epaxial musculature, and the presence or absence of epaxial muscle atrophy. Scoring systems are specified in Table 1. Diagnostic analgesia was not performed.

A routine lameness examination was performed by a diplomate by the European College of Veterinary Surgery (ECVS). This examination aimed to identify horses with limb lameness with or over 2/5 degrees, that were not included in the study. Of the 80 initial horses in the study, according to the criteria established for the clinical examination, 40 were selected for diagnostic imaging.

### 2.3. Diagnostic Imaging

Ultrasonographic and radiographic examinations were performed in the 40 horses selected after clinical examination. A ultrasonography scan of the supraspinous and interspinous ligaments of the back was performed using a linear probe transducer with frequencies of 10 Mhz.

Radiographs were made with the horses standing squarely on all 4 feet to avoid lateroflexion or rotation of the spine. Lateromedial radiographs were obtained from the 10th thoracic to the 1st lumbar vertebra. Findings were evaluated by a specialized radiologist for the presence of spinous process impingement and articular facet pathology. The scoring system used is presented in Table 2 (taken from [6,19].

From the 40 horses subjected to diagnostic imaging, 18 were selected after applying inclusion criteria in relation to the ultrasonographic and radiographic findings. These examinations, consequently, were performed only once and they were not repeated at the end of the experiment.

### 2.4. Procedures Therapies

A device that operates at a frequency of 448 kHz, especially designed for horses, was used (Indiba^®^ Animal Health S.A., Barcelona, Spain). Briefly, this device uses an active electrode and a large flexible metallic plaque as the inactive electrode, which was fixed to the sternal region of the horses. The device delivers radiofrequency energy in two different modes at the active electrode, capacitive (CAP) and resistive (RES). More information about this device can be found in a previous article published by the authors [15].

The CRET group was subjected to a total of four CRET sessions, applied 2 days/week, during 2 consecutive weeks. The number of sessions per week was chosen on the basis of previous clinical reports in human patients with different musculoskeletal disorders, which described improved function and reduced pain when this therapy was applied 2–3 days/week [21,22,23]. Sedation was not needed for the treatment. Each session had a duration of 40 and 20 min for each side of the midline from thoracic T10 to lumbar L6. On each side, therapy consisted of 5 min of CAP therapy at 50%, followed by 12 min of RES therapy at 50% and 3 min of CAP therapy at 12%. The same duration of the intervention was performed in the SHAM group but with the device without power.

### 2.5. Accelerometer and Accelerometric Recordings

A portable 3D gait analyzer (Equimetrix, Centaure-Metrix^®^, Evry, France) was placed on the caudal part of the sternum, between the right and left pectoralis ascendens muscles, using a girth. This location (PECT) is closer to the body center of gravity, and has a good stability against the body of the horse, providing information about the acceleration parameters in the forelimb [15,24,25,26,27]. In order to better analyze the accelerometric parameters in the hindlimbs, the accelerometer was also attached to the sacral midline (SML), with adhesive tape.

This device consists of three orthogonal accelerometers that measure acceleration along the body axis, a data logger and a software program for processing acceleration signals. Recordings of accelerometric data were made in continuous mode with a sampling rate of 100 Hz. Positive data were obtained with accelerations following dorsal, cranial and left directions. This device has been used previously to evaluate gait in horses during racing, jumping or dressage exercises [28,29,30], during an exercise on a water treadmill [27], in lame horses [26,31] or in horses with ataxia induced by the administration of different sedative agents [24,32].

The accelerometric recordings were performed for the 18 horses that followed the inclusion criteria, before and after the therapy. These recordings were always carried out in the same track for all the horses and were led by the same handler, which had experience in keeping the horses at a constant speed. This handler led the horses by hand in a straight line, both at walk and at trot, allowing a free frame of movement and keeping the velocity as constant as possible. A total of 4 runs of 80 m each were recorded for each gait. Only accelerometric measurements were considered for analysis from the runs in which variations were no more than 0.1 m/s at the walk and 0.2 m/s at the trot. In the selected runs, 12 segments of 10 s were measured, avoiding the beginning and the end periods of each run, in order to assure a constant velocity. Data were presented as the means of these 12 measurements. Velocity in each run was measured with a stopwatch.

### 2.6. Accelerometric Parameters

The accelerometric parameters registered by the device were divided into three different groups: energetic, coordination and stride spatiotemporal parameters. Stride energetic parameters measured by the device were total power (TP, W/kg), power into the three body axes (i.e., dorsoventral power DVP, longitudinal power LP and mediolateral power MLP) and dorsoventral displacement (DVD, cm). DVP was calculated by the device as the integral of the power spectrum derived from fast Fourier transformation from the dorsoventral acceleration signal, LP was calculated from the power spectrum obtained from the longitudinal acceleration signal and MLP from the lateral acceleration signal. DVD was calculated as an estimation of the double integration of the dorsoventral acceleration signal. More information about these procedures has already been published [24,25,28,29,30,33]. DVP is considered to measure limb suspension and loading activity, LP measures the craniocaudal or longitudinal activity and MLP measures the amount of acceleration and deceleration along the lateral axis. TP was calculated from the sum of the three body axes’ powers.

Stride coordination parameters measured were regularity (REG, dimensionless) and symmetry (SYM, dimensionless), which measures the similarity of the acceleration pattern of successive strides and the similarities between left and right dorsoventral acceleration patterns, respectively, for both parameters [15,24,26,27,29,32].

From the velocity, stride spatiotemporal parameters obtained by the accelerometer were stride frequency (SF, strides/s or Hz) and length (SL, m). A high correlation has been found in the measurements of these parameters compared to videography [34].

### 2.7. Statistical Analysis

Results of the clinical examination of the back are presented as median and quartiles. Data of the accelerometric parameters are presented as the mean and standard deviation of the percentage of the values relative to the baseline recordings for each intervention group. The normality of the variables was checked with a Shapiro-Wilk’s W test, which showed that these variables did not adjust to a Gaussian distribution. Comparisons between the SHAM and CRET groups were performed with a Mann-Whitney test, whereas comparison between before and after the intervention for each group was carried out with a Wilcoxon matched pair test. In all cases, a value of *p* < 0.05 was considered significant. Statistical study was made with the program Statistic for windows (v. 13.3).

## 3. Results

The results of the examination, before and after the intervention are presented in Table 3. In the thoracolumbar palpation, the SHAM group had the same score before and after the intervention (1 degree), whereas the CRET group presented an improvement of one degree after the intervention. Epaxial muscle pain improved by one degree in the SHAM group and two degrees in the CRET group. There was no significant change in the degree of epaxial atrophy with any intervention (Table 3).

Table 4, Table 5, Table 6 and Table 7 show accelerometric parameters for the walk and trot, and with the accelerometer fixed at PECT and SML. Minor significant changes were detected in the SHAM group after intervention. The only significant change was a decrease in LP at the walk, with the accelerometer at SML. The most consistent changes after CRET intervention was the increased DVP, which was found at the two gaits and with the accelerometer at both positions. LP increased at the walk with the accelerometer at PECT but decreased with the accelerometer at SML. MLP increased at walk with the accelerometer at PECT and at trot, with the accelerometer at SML. TP also increased at walk with the accelerometer at PECT and at trot with the accelerometer at both positions. Minor changes were found in SL, which increased at trot with the accelerometer at SML. Stride REG and SYM only increased significantly at trot with the accelerometer fixed at SML (Table 4, Table 5, Table 6 and Table 7).

## 4. Discussion

A recent survey, comparing the therapeutic intervention of back pain in horses by different European equine clinicians 10 years apart (2006 and 2016) revealed that there has been a marked reduction in the use of intramuscular and intravenous drugs and an increase in the use of local treatments including mesotherapy, injections of the thoracolumbar facets, sacroiliac joint region and injections of the dorsal spinous process’ interspaces [35]. Furthermore, the use of manual therapies and electrophysical agents increased 40%. In this survey, 40, 29 and 22% of the respondents considered osteopathy, kinesiotherapy, and acupuncture useful. However, most of the responders did not use other therapies such as CRET (68%). They had the perception that extracorporeal shock waves induced good or excellent results, but surprisingly only 37% of the responders used this therapy. These results might be partially linked to the scarce scientific studies that have been evaluating the efficacy of different electrophysical agents in specific equine musculoskeletal disorders. These studies in a near future could increase the use of this therapies, as our scientific knowledge about them rises. In fact, recently Trager et al. [6] reported that extracorporeal shock wave therapy resulted in an increased mechanical nociceptive threshold in horses with thoracolumbar pain.

The most remarkable findings of the current clinical study were that the horses of the CRET group, after four treatment sessions, experienced greater increases in DVP, MLP and LP and showed a greater reduction in thoracolumbar and epaxial pain compared to the horses of the SHAM group.

The first part of the first hypothesis proposed in our research was that CRET horses would show higher DVD and DVP compared to the SHAM horses. This hypothesis was formulated based on results from previous studies that have described that horses with back pain had reduced dorsoventral flexion-extension range of movement compared to sound horses [3]. Furthermore, Holm et al. [36] also documented an increased range of motion for dorsoventral movement after an injection of mepivacaine around the interspinous spaces between T16 and L2 in healthy horses. These authors hypothesized that the injection of local anesthetic solution affected the natural rigidity of the back, causing an increased flexibility. Using the same accelerometer that we used in our research, Aujol et al. [17] and Calvo-Santesmases et al. [18] confirmed that the horses with back pain had lower DVD and DVP values. Additionally, in one of these reports [17], the values of both parameters increased in response to an osteopathic treatment. Unfortunately, our data did not completely support this first hypothesis, because we did not find significant changes in DVD in either of the two intervention groups. The authors do not have a clear explanation for the absence of significant variations on DVD. It could be speculated that a more marked clinical improvement would need to be achieved in order to find a significant modification in this parameter, as a consequence of the different sensitivity of the accelerometer to quantify each parameter. DVD values are expressed in cm without decimals, while DVP values are expressed with one decimal number. It would have been of interest to evaluate the horses before the onset of thoracolumbar dysfunction.

The increase in DVP after CRET intervention was a very consistent finding, both at walk and at trot, whereas in the SHAM horses, this parameter did not experience any significant change. DVP, a parameter indicative of the activity along the dorsoventral axis, is a desirable characteristic for dressage and jumping horses, and significant increases have been described in response to age and training [37]. Furthermore, and according to Barrey [25], elasticity, which is a favorable trait in dressage and jumping horses, is partially determined by a greater DVP. A flexible back is required to transmit the forces from a greater hindlimb engagement. Consequently, it can be speculated that the greater DVP after CRET intervention would be the result of an enhanced back flexibility. The increase in DVP in the CRET horses agrees with the results of the physical examination of the back. Both groups of horses, at the beginning of the research, were scored with 2, indicating stiffness in the epaxial musculature with lordosis or movement away from pressure to palpation. After the intervention, the horses of the SHAM group had a mild improvement, but one degree of stiffness of the epaxial musculature remained. On the contrary, after CRET intervention, the horses were scored as 0, reflecting a significant reduction in back stiffness, a result which could have been the reason for the increased DVP.

As a second hypothesis of our research it was proposed that CRET therapy resulted in an increased TP. Our data confirmed this hypothesis, since after the CRET intervention, the horses experienced a significant increase in TP, while the SHAM horses did not. In our opinion, these results could be attributed to a better adaptation to training in the CRET group. An attempt was made to ensure that the trainers maintained the same intensity and duration of the training sessions followed prior to the interventions as much as possible. However, it cannot be completely ruled out that the trainers were subconsciously more conservative with those horses that might continue to have thoracolumbar discomfort, and hence the significant differences in TP. We could also speculate that the horses after CRET therapy could have a greater muscle activity because of an accelerated metabolic activity and improved local circulation and hemoglobin oxygenation, as has been shown in some studies performed in human subjects [10,12]. In fact, we previously found increased TP values during the first 24 h after CRET therapy in healthy horses, which were supposedly attributed to a rise in temperature due to the electromagnetic energy, which triggers the oscillation and friction forces of charged molecules in the tissues [15]. However, in our previous study, we carried out the accelerometric measurements during the first 24 h after CRET intervention, whereas in the current research, the accelerometric examination was performed at least 48 h after the fourth CRET session (mean time: 52.3 ± 5 h; range: 50–58 h). To the authors’ best knowledge, the persistence of increased tissue temperature after CRET therapy has not been investigated for longer than 24 h.

Another kinetic parameter, MLP, increased after CRET intervention, but only at the walk with the accelerometer at PECT and at the trot with the accelerometer at SML. In our opinion, these results were also attributable to the greater flexibility of the back, as happened with DVP. The reason why at trot this was increased in comparison to both gaits, is that naturally at walk the dorsoventral and mediolateral movements are augmented. A surprising finding was the reduction in LP after the CRET and SHAM intervention. In our previous study, the application of CRET therapy resulted in increased values of LP [15]. Likewise, in horses with back pain, submitted to osteopathy, a significant increase in LP was reported [17]. A plausible explanation for our results could be the type of physical activity and training to which the 18 horses of our study were subjected. Barrey and Biau [37] described that, as collection improves, the vertical component of the acceleration increases and simultaneously, the longitudinal component of the acceleration decreases, that is, the TP is used to increase DVP rather than increase LP, and the same would have happened in our horses, regardless of the type of intervention the horses had undergone.

Stride REG and SYM were hypothesized to increase after CRET intervention compared to the SHAM intervention. However, this third hypothesis was rejected. REG and SYM only increased at trot and with the accelerometer at SML. The application of a CRET therapy in the neck, back and croup of healthy horses did not modify these parameters either [15]. It may be hypothesized that CRET therapy does not modify stride REG and SYM when dealing with healthy horses [15], or with back pain but without obvious lameness. It would be interesting in a near future to quantify back kinematics before and after CRET treatments.

In the current study, we included horses with thoracolumbar pain, without an obvious complaint of performance loss and with relatively homogeneous clinical findings which continued in training and in active competition throughout the study. Therefore, the effects of the intervention (SHAM or CRET) did not overlap with a reduction in activity, which could result by itself in changes reflected in the clinical and accelerometric parameters. In addition, and due to the difficulty of standardizing the training program during the study, only horses trained by the same trainers were selected and no changes in training routine were introduced during this period.

Even though our research has some methodological constraints; linked to a clinical study performed with client-owned horses, this is the first study assessing the effects of CRET therapy in horses with thoracolumbar pain. Perhaps the changes observed would have been more marked if the horses recruited for the study would have presented more obvious clinical signs. Some accelerometric parameters including spatio-temporal and energetic parameters can be modified with training [30,37]. Because of this reason, we tried to maintain the same training program during the period of study. Therefore, the accelerometric changes observed could be directly attributed to the intervention and not to the modification of the training program. It is clear that additional standardized studies should be carried out in horses with more marked clinical signs. In addition, the horses were subjected to only four sessions and probably a higher number of sessions would have resulted in more evident accelerometric changes. In fact, between 6 and 10 sessions have been reported to result in improved function and disability and reduced pain in human beings with low back pain [21], myofascial chronic pain [22], knee osteoarthritis [23,38] and shoulder impingement syndrome [39]. 

A second limitation could be that diagnostic anesthesia was not pursued in order to confirm the radiological diagnosis. Overriding dorsal spinous processes is the most common radiographic finding in horses with thoracolumbar pain [5,40]. However, there are variable clinical signs associated with this disorder and a poor correlation between radiographic findings and clinical diseases has been described [5,20,41]. Consequently, many clinicians consider that diagnostic anesthesia is the most definitive method of identifying horses with pain attributable to thoracolumbar disease [5,20,41]. However, Holm et al. [36] demonstrated that both the injection of a mepivacaine solution and sodium chloride in the back resulted in kinematic changes in asymptomatic horses, perhaps due to mechanical–neuromuscular changes in the musculature. Additionally, the potency that we selected for CRET therapy in the present research, that we believed that would have thermal effects, even though subthermal or electrical effects cannot be ruled out. According to Stubbs et al. [4] and Clayton [9], independently of the inciting cause of back pain, they all often lead to a common pathway involving changes in neuromotor control and the neurogenic atrophy of multifidi muscles. This atrophy might result in spinal instability and the micromotion of the joint and consequently, the longissimus dorsi muscle will present stiffness and pain. Therefore, a deep thermal effect would reduce stiffness in spite of the origin of the disease.

A third limitation is that horses belonged to different breeds. Faber et al. [42] evaluated the repeatability of back kinematics in two groups of horses, five Dutch Warmblood horses and five Standardbred trotters, studied in two different laboratories. Most differences between both groups were found at trot, with a significantly larger range of motion values found in Standardbred trotters for flexion–extension in T10, T13 and L5, for lateral bending in T13 and T17 and for protraction and retraction. The authors associated these results with the fact that, after normalization for height at the withers, the two groups of horses appeared to use a different combination of SL and stride duration to achieve the required velocity. According to Faber et al. [42], it was probable that these differences were caused by between-breed differences rather than by between-laboratory differences. Furthermore, Barrey et al. [29] compared the walk, trot and conformation in three breeds of dressage horses, German, French and Spanish saddle horses, tested with the accelerometric gait analysis system Equimetrix and they found that many gait variables were significantly different between breeds.

Another limitation is that the changes in superficial and deep temperature in the treated tissues were not measured. The skin temperature could have been measured by thermography. However, the most interesting point would be to measure the increase in temperature in the epaxial musculature. To do this, it would have been necessary to surgically implant thermal probes inside the muscles. The evaluation of client-owned sport horses precludes these measurements.

The combined evaluation of the results found in the current study together with the data provided in a previous report performed by our research team [15] appears to show that the application of this therapy might to be beneficial in sport horses. However, the exact time after a training session or competition and the number and frequency of CRET sessions should be strictly defined in the future, both in sound horses and those with musculoskeletal disorders. Of special relevance is the evaluation of the application of CRET at specific times after a training session. It should be experimentally verified whether the application of CRET immediately after exercise could limit the cellular adaptations to exercise, in association with a possible modulation of the minor exercise-induced injury, which is necessary to improve performance, as established by the training principle of supercompensation. In 14 trained male runners performing two incremental treadmill running tests two days apart after an exhaustive training session, Duñabeitia et al. [43] found that when the athletes were subjected to a CRET session 2 h after intense exercise, the treated group presented greater increases in stride length, angle and height between the first and second tests than the control group. Theses authors concluded that the CRET intervention was applied 2 h after intense training enhanced the biomechanical parameters compared to passive rest, generating a more efficient running pattern. This type of research has not yet been carried out in the sport horse, but it deserves future studies.

## 5. Conclusions

Applications of four sessions of a CRET radiofrequency at 448 kHz during two consecutive weeks, in nine horses with thoracolumbar pain induced a consistent increase in DVP at walk and at trot, which was not observed with the same therapy when it was applied to another nine horses, but with the device without power (SHAM intervention). Other accelerometric changes were increased TP and MLP. All of these changes appear to suggest an increase grade in flexibility of the back, which was in accordance with the results of the physical examination after the treatments. A reduction of 1 degree in thoracolumbar pain and in 2 degrees in epaxial muscle pain was also found after CRET therapy. On the other hand, after SHAM intervention, only an improvement of 1 degree in thoracolumbar pain was detected. These results might suggest that the application of CRET therapy could be a beneficial strategy to improve the locomotion and back flexibility in horses with mild to moderate thoracolumbar pain.

## Figures and Tables

**Table 1:** Scoring of back examination, considering thoracolumbar palpation over the spinous processes, epaxial muscle palpation and the assessment of longissimus dorsi muscle atrophy (modified from [6,19]).

Scoring	0	1	2	3
**Thoracolumbar Palpation over the Spinous Processes**				
On midline, directly over the spinous processes from the withers to the tuber sacrale	No obvious avoidance	Avoidance and lordosis of spine or movement away from pressure	Avoidance plus tossing of head and/or tail swish	Violent avoidance characterized by kicking or biting
**Epaxial Musculature Palpation**				
5–10 cm off midline, abaxial to the spinous processes	Soft musculature with no avoidance	Stiff musculature with no avoidance	Stiff musculature with avoidance characterized by lordosis or movement away from pressure	Stiff musculature with violent avoidance characterized by kicking or biting
**Epaxial Muscle Atrophy**				
Visual examination	No atrophy	Atrophy in the thoracic or lumbar region	Atrophy in the thoracic and lumbar region	

**Table 2:** Scoring of the radiographic findings (taken from [6,20].

Score	Description	Number of Horses
0	No abnormalities noted	0
1	Narrowing of interspinous space (<4 mm), mild sclerosis of cortical margins	14
2	Loss of interspinous space, moderate sclerosis of cortical margins	4
3	Loss of interspinous space, severe sclerosis of cortical margins, osteolysis of spinous processes	0
4	Loss of interspinous space, severe sclerosis of cortical margins, osteolysis and cystic formation of spinous processes	0

**Table 3:** Median and quartiles of back examination in 9 horses subjected to 4 capacitive resistive electric transfer (CRET) during 2 consecutive weeks compared with 9 horses subjected to a SHAM procedure (machine with the power off).

Intervention	Control	Thoracolumbar Examination	Epaxial Musculature	Epaxial Musculature Atrophy
SHAM	Baseline	1 (1–2)	2 (2–2)	0 (0–1)
	After	1 (0–1) n.s.	1 (1–2) *	0 (0–1) n.s.
CRET	Baseline	1 (1–1)	2 (1–2)	0 (0–1)
	After	0 (0–1) *	0 (0–1) *	0 (0–1) n.s.

(n.s.: non-significant differences between baseline and after intervention for SHAM and CRET groups) (*: significant differences between baseline and after intervention for SHAM and CRET groups).

**Table 4:** Mean values of the accelerometric parameters measured at walk and with the accelerometer fixed at the sternum region, before and after the intervention (SHAM group and capacitive resistive electric transfer therapy group (CRET)). Data are expressed as the mean percentage ± SD, relative to baseline values.

Accelerometric Parameters	SHAM Intervention		CRET Intervention	
	Baseline	After	Baseline	After
DVD (cm)	100 ± 0.0	95.2 ± 21.7	100 ± 0.0	95.8 ± 18.5
DVP (W/kg)	100 ± 0.0	98.9 ± 28.9	100 ± 0.0	112.6 ± 26.1 * A
LP (W/kg)	100 ± 0.0	96.2 ± 25.9	100 ± 0.0	121.0 ± 28.8 * A
MLP (W/kg)	100 ± 0.0	109.2 ± 28.7	100 ± 0.0	129.8 ± 32.6 * A
TP (W/kg)	100 ± 0.0	100.8 ± 23.9	100 ± 0.0	121.6 ± 23.0 * A
REG (dimensionless)	100 ± 0.0	100.0 ± 20.1	100 ± 0.0	95.9 ± 28.8
SYM (dimensionless)	100 ± 0.0	111.6 ± 45.5	100 ± 0.0	94.6 ± 33.7
V (m/s)	100 ± 0.00	98.72 ± 5.73	100 ± 0.00	102.1 ± 7.25
SF (strides/s)	100 ± 0.0	97.8 ± 4.7	100 ± 0.0	102.9 ± 5.8
SL (m/stride)	100 ± 0.0	100.9 ± 5.7	100 ± 0.0	99.5 ± 5.7

(* significantly different from baseline in each intervention group; A: significantly different between group SHAM and CRET after intervention) *p* < 0.05. (DVD: dorsoventral displacement; DVP: dorsoventral power; LP: longitudinal power; MLP: mediolateral power; TP: total power; REG: stride regularity; SYM: stride symmetry; V: velocity; SF: stride frequency; SL: stride length).

**Table 5:** Mean values of the accelerometric parameters measured at walk and with the accelerometer fixed in the sacrum midline, before and after the intervention (SHAM group and capacitive resistive electric transfer therapy group (CRET)). Data are expressed as the mean percentage ± SD, relative to baseline values.

Accelerometric Parameters	SHAM Intervention		CRET Intervention	
	Baseline	After	Baseline	After
DVD (cm)	100 ± 0.0	93.4 ± 15.8	100 ± 0.0	108.2 ± 13.3
DVP (W/kg)	100 ± 0.0	94.1 ± 25.9	100 ± 0.0	115.0 ± 14.3 * A
LP (W/kg)	100 ± 0.0	88.7 ± 18.2 *	100 ± 0.0	88.7 ± 19.9 *
MLP (W/kg)	100 ± 0.0	99.9 ± 22.2	100 ± 0.0	95.8 ± 17.3
TP (W/kg)	100 ± 0.0	92.8 ± 19.2	100 ± 0.0	95.6 ± 10.2
REG (dimensionless)	100 ± 0.0	109.6 ± 26.4	100 ± 0.0	113.4 ± 15.4
SYM (dimensionless)	100 ± 0.0	118.3 ± 24.3	100 ± 0.0	113.0 ± 17.3
V (m/s)	100 ± 0.00	97.15 ± 8.35	100 ± 0.00	101.9 ± 8.85
SF (strides/s)	100 ± 0.0	99.7 ± 8.6	100 ± 0.0	101.5 ± 5.7
SL (m/stride)	100 ± 0.0	97.6 ± 8.4	100 ± 0.0	101.6 ± 14.6

(* significantly different from baseline for each parameter within each intervention group; A: significantly different for each parameter between group SHAM and CRET after the intervention) *p* < 0.05. (DVD: dorsoventral displacement; DVP: dorsoventral power; LP: longitudinal power; MLP: mediolateral power; TP: total power; REG: stride regularity; SYM: stride symmetry; V: velocity; SF: stride frequency; SL: stride length).

**Table 6:** Mean values of the accelerometric parameters measured at trot and with the accelerometer fixed at the sternum region, before and after the intervention (SHAM group and capacitive resistive electric transfer therapy group (CRET)). Data are expressed as the mean percentage ± SD, relative to baseline values.

Accelerometric Parameters	SHAM Intervention		CRET Intervention	
	Baseline	After	Baseline	After
DVD (cm)	100 ± 0.0	101.8 ± 16.0	100 ± 0.0	103.5 ± 10.2
DVP (W/kg)	100 ± 0.0	102.5 ± 12.5	100 ± 0.0	117.4 ± 12.4 * A
LP (W/kg)	100 ± 0.0	93.4 ± 20.9	100 ± 0.0	94.3 ± 19.4
MLP (W/kg)	100 ± 0.0	97.9 ± 18.1	100 ± 0.0	101.8 ± 15.6
TP (W/kg)	100 ± 0.0	98.2 ± 14.1	100 ± 0.0	121.2 ± 13.2 * A
REG (dimensionless)	100 ± 0.0	101.3 ± 17.79	100 ± 0.0	99.8 ± 14.4
SYM (dimensionless)	100 ± 0.0	105.3 ± 16.0	100 ± 0.0	104.8 ± 15.9
V (m/s)	100 ± 0.00	96.3 ± 9.60	100 ± 0.00	101.4 ± 15.22
SF (strides/s)	100 ± 0.0	99.6 ± 4.18	100 ± 0.0	100.7 ± 3.9
SL (m/stride)	100 ± 0.0	97.03 ± 8.30	100 ± 0.0	117.7 ± 12.5 * A

(* Significantly different from baseline in each intervention group; A: Significantly different between group SHAM and CRET after intervention) *p* < 0.05. (DVD: dorsoventral displacement; DVP: dorsoventral power; LP: longitudinal power; MLP: mediolateral power; TP: total power; REG: stride regularity; SYM: stride symmetry; V: velocity; SF: stride frequency; SL: stride length).

**Table 7:** Mean values of the accelerometric parameters measured at trot and with the accelerometer fixed in the sacrum midline, before and after the intervention (SHAM group and capacitive resistive electric transfer therapy group (CRET)). Data are expressed as the mean percentage ± SD, relative to baseline values.

Accelerometric Parameters	SHAM Intervention		CRET Intervention	
	Baseline	After	Baseline	After
DVD (cm)	100 ± 0.0	95.6 ± 15.07	100 ± 0.0	95.99 ± 13.5
DVP (W/kg)	100 ± 0.0	93.6 ± 18.8	100 ± 0.0	121.6 ± 14.0 *
LP (W/kg)	100 ± 0.0	101.3 ± 15.2	100 ± 0.0	101.6 ± 22.3
MLP (W/kg)	100 ± 0.0	102.7 ± 17.4	100 ± 0.0	132.9 ± 18.2 * A
TP (W/kg)	100 ± 0.0	98.7 ± 17.5	100 ± 0.0	128.5 ± 19.4 * A
REG (dimensionless)	100 ± 0.0	101.9 ± 12.7	100 ± 0.0	123.5 ± 17.6 *A
SYM (dimensionless)	100 ± 0.0	102.3 ± 21.5	100 ± 0.0	120.9 ± 21.5 *A
V (m/s)	100 ± 0.00	96.14 ± 7.74	100 ± 0.00	103.1 ± 9.3
SF (strides/s)	100 ± 0.0	100.5 ± 4.4	100 ± 0.0	101.7 ± 4.5
SL (m/stride)	100 ± 0.0	95.5 ± 7.3	100 ± 0.0	101.2 ± 9.2

(* significantly different from baseline in each intervention group; A: significantly different between group SHAM and CRET after intervention) *p* < 0.05. (DVD: dorsoventral displacement; DVP: dorsoventral power; LP: longitudinal power; MLP: mediolateral power; TP: total power; REG: stride regularity; SYM: stride symmetry; V: velocity; SF: stride frequency; SL: stride length).
